# Examining *in vivo* effects of silychristin, a potent *in vitro* inhibitor of thyroid hormone transporter MCT8

**DOI:** 10.3389/ftox.2026.1796387

**Published:** 2026-04-21

**Authors:** Kostja Renko, Ryne Thomas, MaryAnn Hawks, Jermaine Ford, Josef Köhrle, Marta Axelstad, Mary E. Gilbert

**Affiliations:** 1 BFR German Federal Institute for Risk Assessment, German Centre for the Protection of Laboratory Animals (Bf3R), Berlin, Germany; 2 Office of Research and Development, Environmental Protection Agency, Research Triangle Park, Durham, NC, United States; 3 Oak Ridge Institute for Science Education Oak Ridge, Oak Ridge, TN, United States; 4 Charité Universitätsmedizin Berlin, Corporate Member of Freie Universität Berlin, Humboldt-Universität zu Berlin, Institut für Experimentelle Endokrinologie, Berlin, Germany; 5 National Food Institute, Technical University of Denmark, Lyngby, Denmark

**Keywords:** *in vivo*, Mct8, silybin, silychristin, thyroid hormone, thyroid hormone transmembrane transporter, deiodinase, dehalogenase

## Abstract

Thyroid hormone (TH) availability is particularly critical for early brain development. TH transport across the blood-brain barrier is facilitated through two main transmembrane transporters: monocarboxylate transporter 8 (MCT8) and organic anion transporter 1C1 (OATP1C1). Inhibition of MCT8-mediated TH transport has been identified for a number of environmental chemicals using *in vitro* screening assays. Here we examined the *in vivo* effects of exposure to a potent *in vitro* inhibitor of MCT8, the flavonolignan silychristin, on several aspects of the TH system. Adult female rats were daily gavaged with 0, 250, or 500 mg/kg/day (n = 10/group) of silychristin for 7 days and euthanized on day 8. A smaller group (n = 5/group) of rats was administered the related flavonolignan, silybin (900 mg/kg), or the milk-thistle-derived flavonolignan mixture, silymarin (1,500 mg/kg). Serum TH concentrations were not changed in any treatment group. *Mct8* and *Oatp1c1* expression were upregulated in the choroid plexus upon silymarin exposure, without change in response to silychristin or silybin. Deiodinase 1 and dehalogenase activities, unchanged in the liver, were increased in the thyroid by the high dose of silychristin. These changes may have been triggered by increased thyroidal TH content, consequent to a reduction in MCT8-mediated TH efflux. Pharmacokinetic properties of silychristin and other flavonoids result in their low bioavailability and likely contributed to the largely negative findings. These observations demonstrate the challenges in extrapolating results from *in vitro* models to studies in intact organisms, showcasing the importance of selecting appropriate animal models and the best experimental design for assessing effects on human health.

## 1 Introduction

Thyroid hormones (TH) strongly influence the homeostatic regulation of energy metabolism, cardiac function, growth, and differentiation in the adult organism and are crucial regulators of early developmental processes (Mullur et al., 2014). TH availability is especially critical for early brain development (Garcia-Aldea et al., 2024) and is facilitated by active transport from blood to brain through two main transmembrane transporters: monocarboxylate transporter 8 (MCT8) and organic anion transporter 1C1 (OATP1C1). In the immature mouse and rat brain, MCT8 mRNA and protein are strongly expressed in cerebral microvessels, neurons, endothelial cells, and barrier structures but significantly decline with age (Wilpert et al., 2020). In the adult rat and human brain, MCT8 is most concentrated on the epithelial cell apical surface of the choroid plexus (Roberts et al., 2008). Transport of TH across cell membranes combined with the intracellular activation and inactivation of THs by deiodinase enzymes (DIO) provides exquisite control of TH action at the cellular level. The prominent role of MCT8 to supply TH to the developing human brain was established when genetic mutations of the *SLC16A2* gene, which codes for MCT8, were discovered to underlie Allen-Herndon-Dudley Syndrome (AHDS), a severe neurological condition characterized by developmental delays and psychomotor impairments (Garcia-Aldea et al., 2024). AHDS patients present with low free T4 and regular or elevated free T3 concentration in blood, while the brain may suffer a “hypothyroid” status (Bernal et al., 2015). Mouse models of genetic inactivation of *Mct8* share some of these alterations in TH blood levels but lack several of the respective neurological impairments. However, when combined with removal of other TH transmembrane transporters or TH metabolizing enzymes, several neurological aspects of the human syndrome are recapitulated including delayed myelination, oligodendrocyte maturation, and alterations in neurogenesis (Garcia-Aldea et al., 2024; Maity-Kumar et al., 2022; Alevyzaki et al., 2025).

The complexity of TH signaling presents a variety of potential sites for chemical interference that can disrupt TH regulation and function (Capen, 1994; Köhrle and Frädrich, 2021; Gilbert et al., 2020; Thambirajah et al., 2019). *In vitro* assays have verified the activity of environmental chemicals at several of these sites and, recently, several reports have demonstrated chemical interference with MCT8-mediated TH transport (Jayarama-Naidu et al., 2015; Dong and Wade, 2017; Wagenaars et al., 2024). In these studies, the flavonolignan silychristin, a natural compound isolated from milk thistle extract, was identified as a potent and specific inhibitor of cellular MCT8-mediated TH uptake (Johannes et al., 2016). A selective action of silychristin on MCT8 was confirmed in transgenic cells expressing the human MCT8 and in murine primary astrocytes, displaying comparable potencies across species and platforms. Recent characterization of its molecular binding properties to the transporter has cemented silychristin as an optimal reference compound for *in vitro* test model systems (Chen et al., 2022; Tan et al., 2025).

Augmented by concern for neurodevelopmental sequelae, verification and translation of *in vitro* screening outcomes to hazard characterization of environmental chemicals in rodent models is still an essential component of toxicological assessment. The fidelity of translation of both simple screening level assays as well as more complex human-based *in vitro* models must be verified to increase confidence in their application and use in risk-based human health decision making (Foley et al., 2024; Fritsche et al., 2021). Despite clear evidence of neurodevelopmental impairments resulting from genetic mutation in humans and manipulation of expression of TH transmembrane transporters in rodent models, the toxicological significance and signature pattern of ‘chemically induced’ interference with these transporters has yet to be examined. To begin to address this question we sought to determine if the *in vivo* administration of this potent and specific TH transport inhibitor could lead to disruption of the TH system in the intact organism. Although developmental neurotoxicity is of primary concern, we began with a short-term dose-range finding study in young adult female rats, largely necessitated by the difficulty in procuring sufficient quantities of silychristin for developmental exposures and its high cost. As MCT8 is expressed in the brain, liver, and thyroid gland, silychristin may directly inhibit TH transport to the brain while also dysregulating the hormonal axis through its action in the periphery with secondary consequences to brain TH. We assessed the functional interference of impaired TH transport by comparing silychristin, a pure compound and the most potent MCT8 *in vitro* inhibitor known to date, to silybin and silymarin. Silybin exhibits much less potent MCT8-inhibiting properties; silymarin, meanwhile, is a complex mixture of flavonoids with an undefined portion of silychristin and silybin and widely available ‘hepatoprotective’ over-the-counter food supplement (Johannes et al., 2016; Kren and Valentova, 2022; EMA/HMPC, 2018; Zhao et al., 2024). Serum TH concentrations were investigated as a readout of potential systemic effects of altered transporter function as reported in genetic models. We examined the central action of MCT8 inhibition through TH-dependent gene transcription in the choroid plexus. In the periphery, local effects of MCT8 inhibition were determined by examining thyroidal peroxidase, deiodinase, and dehalogenase in the thyroid gland and liver.

## 2 Methods

### 2.1 *In vivo* study design

Adult female Long–Evans rats (40 -50 days of age) were obtained from Charles River (Kingston, New York) and pair housed in standard plastic hanging cages in an approved animal facility. All experiments were conducted with prior approval from the US EPA’s Institutional Animal Care and Usage Committee (IACUC) and were carried out in an Association for Assessment and Accreditation of Laboratory Animal Care (AAALAC)-approved facility. Temperature- and humidity-controlled animal rooms were maintained on a 12:12 light:dark schedule and animals were permitted free access to food. On arrival, animals were weighed and assigned to one of five dose groups, balanced by weight, and given free access to water and standard laboratory rodent chow (Purina 5220). One week after arrival, animals were gavaged daily with vehicle (corn oil, n = 10), 250 (n = 10) or 500 (n = 10) mg/kg/day silychristin (Ambeed 98% purity, Cas # 33889-69–9, MW 482.44), 900 mg/kg/day (n = 5) silybin (98% purity Ambeed Cas # 42110-65–6, MW 482.44), or 1,500 mg/kg/day (n = 5) silymarin mixture containing 44.7% silybin (Sigma-Aldrich Batch # BCBW6692)... Dose levels and duration of exposure were guided by literature reports summarized by the European Medicines Agency (2016) but were largely determined by the limited quantity available and cost prohibitive supply of silychristin. Dosing solutions were prepared as suspensions in corn oil and kept on stirplate constantly prior to and during dosing procedures. All suspensions were delivered in a 5 mL/kg volume by oral gavage between 8:00 and 10:00 a.m. for seven consecutive days.

### 2.2 Assessment of applied dosing solutions for MCT8 inhibition *in vitro*

MDCK1-hMCT8 were cultivated in DMEM medium (10% FCS, penicilin/streptomycine and G418 as selection antibiotic) and 50.000 cells were seeded in microtiter format (TTP, Switzerland) 2 days prior to testing. On the day of testing, cells were washed with phosphate-buffered saline (PBS) and 40 µL of uptake buffer was added to each well. *In vitro* testing of the applied dosing preparations was conducted as previously described (Johannes et al., 2016). Similar volumes (20 µL) of the dosing material from all treatment groups were diluted in DMSO (180 µL) and vigorously vortexed to ensure distribution of material between the oily preparations (corn oil base of the dosing solutions) and DMSO. For the highest concentration used in the assay, 4 µL of the DMSO-diluted material was further diluted in 196 µL of uptake buffer. Serial dilutions (4-fold) of the dosing materials (corn oil or low and high silychristin, silybin, and silymarin) were prepared, together with a dilution series with known silychristin concentrations from another source (Sigma-Aldrich) as a positive control. Respective dilutions (10 µL) were added to the wells and uptake was started with 50 µL/well of T3 (10 µM) in uptake buffer. The seeded plates were incubated for 1 h at 37 °C, followed by two washing steps with PBS and fridge-cold ddH2O. 50µL of APS solutions (0.6 M in ddH2O) was added to each well, plates were sealed with PCR cover tape, and the plate was incubated for 1 h at 85 °C–90 °C in a convection oven. Plates were cooled in a refrigerator, spun down at 1000g (2 min, RT, Eppendorf), and then 150 µL ddH2O was added to each well. Lysate was further diluted (12.5 µL lysate +37.5 µL ddH2O) and relative iodine content, representing the amount of taken up T3, was quantified via Sandell-Kolthoff reaction. Samples with solvent were used as 100% control, while wells with 50 µM SC served to define full inhibition (0% MCT8 activity). Each condition was tested in two technical replicates.

Effects on cell viability were determined by applying the identical exposure scheme and using ATP-dependent luciferase activity as a viability indicator (Celltiter Glo 2.0, Promega) using a luminescence plate reader (Mithras, Berthold). Medium was removed following incubation, cells were washed, and 50 µL of PBS was added to the cells, followed by dispensing of 50 µL Celltiter Glo solution. Plates were placed on a shaker for 10 min. ATP-dependent luminescence was measured and used as an indicator for cell viability.

### 2.3 Tissue collection

Animals were euthanized by decapitation without anesthesia 24 h after the last dose. Trunk blood was collected, allowed to clot on ice for 30 min, centrifuged, and serum collected for TH analysis. Because MCT8 mediates TH efflux in the thyroid gland and TH uptake in the liver, these organs were collected at the time of euthanasia, weighed, and flash frozen for *ex vivo* assessment of activity of dehalogenase and deiodinase metabolizing as readouts for altered intracellular TH levels. Whole brains were removed from the skull and rinsed in cold PBS. The choroid plexus was collected from the lateral and 4^th^ ventricles and saved in RNAlater™ (Invitrogen) according to the manufacturer’s recommendation. All samples were stored at −80 °C until analysis.

### 2.4 Quantification of thyroid hormones in the serum

TH concentrations were measured in serum by Liquid Chromatography Mass Spectrometry (LCMS) using an AB Sciex (Framingham, MA) Exion AC UHPLC-Qtrap 6,500+ Linear Ion Trap LC/MS/MS system as previously described (Gilbert et al., 2022). Two ion transitions were monitored for each target analyte, qualitatively identified based on retention time relative to the internal (^13^C_6_T3 and ^13^C_6_T4) and calibration standards and the ratio of the peak areas of the monitored ion transitions. Solid phase extraction (SPE) was conducted using Evolute CX 1 mL 10 mg SPE cartridges (Biotage, Charlotte, NC) conditioned with methanol and 0.1% formic acid. Each sample batch consisted of a method blank, a laboratory control sample (blank spike), and a continuing calibration verification sample prepared in solvent. The LLOQ for serum T3 and T4 was 0.01 ng/mL.

### 2.5 Tissue processing of liver and thyroid and measurement of deiodinase and dehalogenase activities

Frozen thyroid lobes collected from each animal were processed as previously described (Renko et al., 2022). Glands were allowed to thaw, suspended in 230 µL Tris-HCl (10 mM, pH7), and homogenized via electronic pestle mixer. Part of the homogenate was saved for protein quantification, iodine content, and peroxidase activity measurements. Another aliquot of 100 µL was mixed with a similar volume of homogenization buffer and used to determine DIO1 and DEHAL1 activities. Liver tissue from each animal was powdered in a frozen state in a bead mill (Mixer CryoMill Retsch) to generate homogenous material for subsequent processing. A dollop of powdered liver collected on the tip of a frozen spatula was mixed with 400 µL homogenization buffer (250 mM D-Sucrose, 20 mM Hepes, 1 mM EDTA, pH 7.4), suspended by vigorous pipetting, and further disrupted by ultrasonic treatment (2 X 10 pulses, 100% amplitude, UP50H, Hielscher, Teltow, Germany).

Protein concentrations of thyroid gland and liver homogenates were determined by BCA assay (Smith et al., 1985) and enzyme activity was determined according to procedures described in Kadic et al. (2024). Homogenates were diluted to a protein concentration of 0.5 μg/μL (thyroid) or 2 μg/μL (liver). For DEHAL1 activity, 20 µg of thyroid or 80 µg of protein was mixed with cofactors (NADPH, FAD) and substrate (10 µM 3-Iodo-L-tyrosine, MIT, Tokyo Chemical Industry). Two liver samples fell below 2 μg/μL protein levels and activity values were respectively adjusted. For DIO1 activity, 20 µg of thyroid or 40 µg of liver was mixed with a respective mixture of buffer and cofactors (0.1 M KPO4, 1 mM EDTA, 40 mM DTT, pH6.8) and rT3 as substrate (10 µM). A fully inhibited sample pool was also prepared for both enzymes to subtract background by adding a high concentration of the DIO1 inhibitor propyl-6-thiouracil (PTU, 1 mM) or the DEHAL1 inhibitor dibromotyrosine (DBT, 1 mM) (Tokyo Chemical Industry). Reaction mixtures were incubated over 2 (DIO1) or 4 h (DEHAL1) at 37 °C under constant shaking (incubated microplate shaker, VWR). Released iodine was eluted via DOWEX 50WX2-loaded columns after adding 100 µL of acetic acid (10% in ddH2O). Eluted iodine was quantified photometrically by utilizing the Sandell-Kolthoff reaction, following the iodine-depending destaining of Ce^+4^ (yellow) to Ce^+3^ (transparent) within its redox-reaction with arsenite. Extrapolation of photometric measurements to absolute activities in “pmol (released iodine)/mg (protein) * min” was done using a separate iodine standard curve. Each sample was tested in two technical replicates.

### 2.6 Relative iodine content and peroxidase activity from thyroid tissue

To monitor any possible interference of flavonolignan with thyroidal iodine homeostasis or TH synthesis, iodine content and peroxidase activity were assessed in thyroid tissue. The relative iodine content was determined by mixing 20 µL of thyroid homogenate (in 10 mM Tris-HCl, 0.5 μg/μL) with 80 µL ammonium persulfate (APS, 0.6 M) in PCR reaction tubes and heating to 80 °C–85 °C in a thermocycler (Eppendorf). Subsequently, digested lysate was 20-fold diluted with ddH2O and a volume of 50 µL was measured photometrically in the Sandell-Kolthoff reaction. Change of absorption after 20 min (dOD) was directly used as a relative measure of the iodine content.

Relative thyroidal peroxidase was taken as an index of activity of the primary synthesis enzyme thyroperoxidase (TPO) and assessed by adding 10 µL of thyroid homogenate (0.5 μg/μL) to a black microtiter plate. Following the addition of 80 µL mastermix (f.c. 0.1 M KPO4, 100 µM Amplex Ultrared), the reactions were started by quickly dispensing 10 µL of H2O2 (0.035%) into each plate and incubating for 20 min at 37 °C under constant shaking (200 rpm). The fluorescence signal for each sample was then measured in a GENios plate reader (TECAN) using a respective filter set (Ex.535 nm/Em.590 nm).

### 2.7 Gene expression in choroid plexus by quantitative real time-PCR

Gene expression was assessed according to standard procedures as previously described in Gilbert et al., 2024 (Gilbert et al., 2024). Total RNA was extracted *via* TRIzol® (Invitrogen) according to the manufacturer’s protocol. RNA pellets were resuspended in nuclease-free H2O, and RNA concentrations were measured on Nanodrop 1,000 (Nanodrop Technologies). RNA samples were treated with DNase I (Promega, M6101) and quantified using the Ribogreen Quantitation Kit (ThermoFisher, R11490). DNase I-treated RNA was reverse transcribed with the ABI cDNA Archive Kit (ThermoFisher, 4322171) and 25 ng equivalent cDNA was amplified in a 12 µL volume using ABI TaqMan Gene Expression Assays and ABI Universal Master Mix (ThermoFisher, 4304437). Amplification was performed on an ABI model 7900HT sequence detection system using standard Taqman cycling parameters. All samples were run in technical duplicate. Choroid plexus was assessed for TH transmembrane transporters (*Mct8, Mct10,* and *Oatp1c1*), distributor protein (*Ttr*), and metabolizing enzymes (*Dio1, Dio2,* and *Dio3*). All Taqman probes used for these analyses are summarized in Table 1. Beta-2-microglobulin (*B2m*) and β-*actin* were used as the endogenous control as neither showed a significant change between control and treated groups by single factor ANOVA. Data were analyzed by relative gene quantification using the 2^ddCt^ method (Livak and Schmittgen, 2001).

**TABLE 1:** Gene probes examined in choroid plexus 24 h after the final dose.

Gene symbol	Gene name	TaqMan assay ID
*Dio1*	Deiodinase 1	Rn00572183_m1
*Dio2*	Deiodinase 2	Rn00581867_m1
*Dio3*	Deiodinase 3	Rn00568002_s1
*Ttr*	Transthyretin	Rn00572183_m1
*Slc16a2 (Mct8)*	Solute carrier family 16 member 2	Rn00596041_m1
*Slco1c1 (Oatp1c1)*	Solute carrier organic anion transporter family Member 1C1	Rn00584891_m1
*Slc16a10 (Mct10)*	Solute carrier family 16 Member 10	Rn00684732_m1
*β2m (Reference Gene)*	β-2 microglobulin	Rn00560865_m1

### 2.8 Statistical analysis

All data were expressed as mean ± standard error mean (SEM). Statistical significance of treatment effects on body weight, serum TH concentrations, enzyme activity, and gene expression were assessed with one-way analysis of variance (ANOVA) using SAS Version 9.2 (Cary, NC) or Prism (Boston, MA). When a significant treatment effect was detected (p < 0.05), pairwise difference among groups were tested *post hoc* using Dunnett adjustment of multiple comparisons. Mean fold-change in gene expression were set at 1.25-fold change and a 5% false discovery rate. To control for experiment-wise error, alpha was reduced to 0.018 by dividing 0.05 by the square root of number of targets examined.

## 3 Results

**FIGURE 1:**
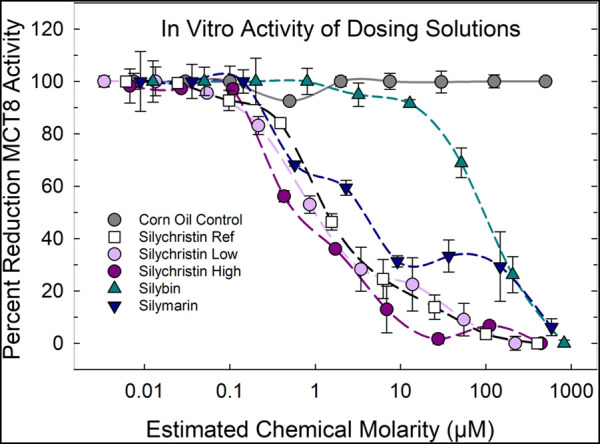
Means (±SE) percent inhibition of MCT8 activity in response to application of dosing solutions delivered to animals in corn oil control, silychristin (250 and 500 mg/kg), silybin (900 mg/kg), and silymarin mixture (1,500 mg/kg). Molar concentrations were estimated and contrasted to silychristin reference. Results indicated similar potencies for silychristin dosing solutions and silychristin reference and significant but less potent effects of silymarin followed by silybin.

To verify and compare the potency of administered solutions in the animal study, aliquots of dosing solutions were tested for an *in vitro* MCT8-inhibition. Application of the corn-oil preparations of silychristin, silybin, and silymarin applied in a concentration-dependent manner to an established MCT8 *in vitro* assay revealed the expected inhibitory potential of these preparations, with silybin being the least efficacious and silychristin solutions closely approximating silychristin positive control reference. Silymarin dosing solutions were less efficacious but tracked a similar profile as silychristin. The *in vitro* potency of the two silychristin dosing solutions, expressed as µM concentrations, were comparable to each other (Figure 1).

**FIGURE 2:**
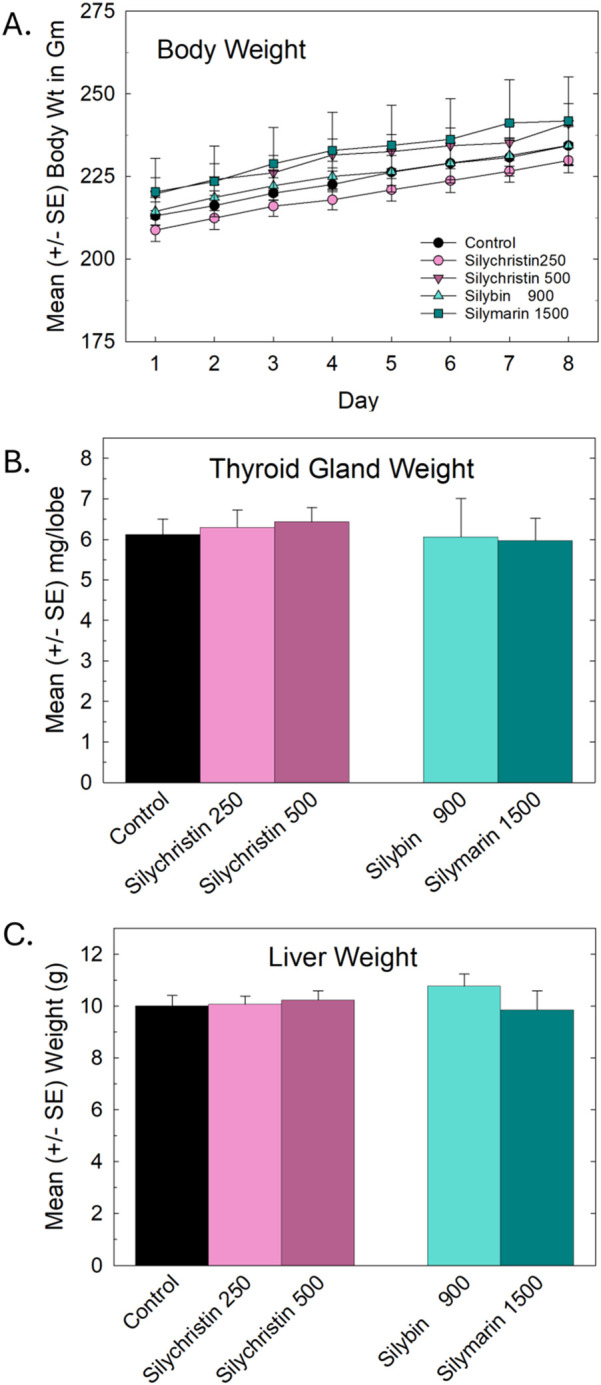
**(A)** Repeated daily dosing with silychristin, silybin, or the silymarin mixture did not alter body weight (Mean ± SE g) **(B)**, thyroid gland weight (mg/lobe), or **(C)** liver weight (g).

**FIGURE 3:**
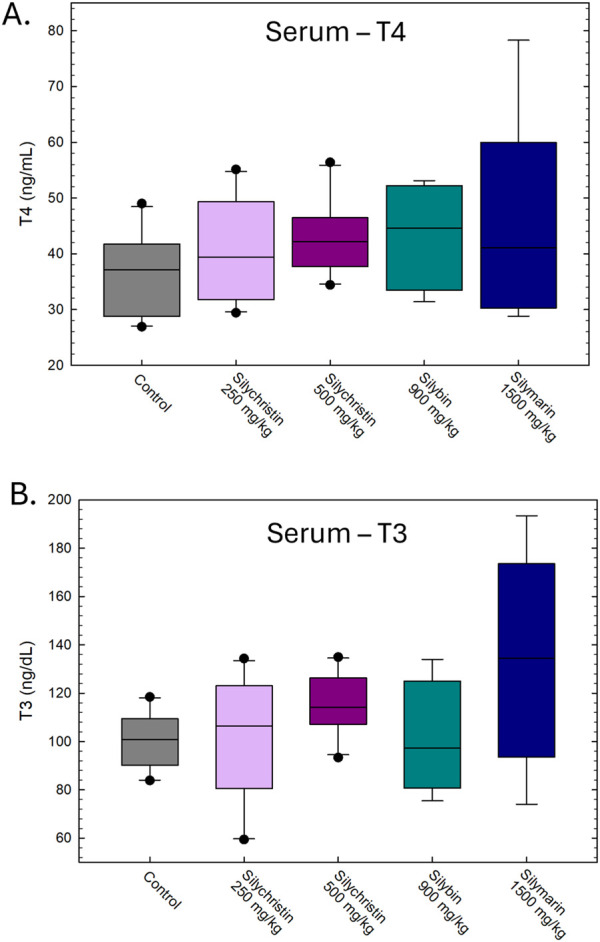
Mean (±SE) serum thyroid hormone concentrations were not changed. **(A)** Serum T4 concentration did not differ among groups dosed with corn oil (n = 10), 250 mg/kg silychristin (n = 10), 500 mg/kg silychristin (n = 10), silybin (n = 4), or the silymarin mixture (n = 4) [F(4,34) = 0.71, p = 0.59]. **(B)** Serum T3 concentration was also not significantly changed [F(4,34) = 2.27, p = 0.0828].

Daily dosing with these solutions did not result in any overt signs of toxicity as evidenced by body, thyroid gland, or liver weights (Figures 2A–C). Nor were any significant differences in total T4 or T3 serum concentrations collected 24 h after the final dose (Figure 3). One animal from the silybin group died from gavage dosing, unrelated to treatment. Higher standard error was evident in the silybin and silymarin groups, partially attributable to small sample sizes (n = 4-5).

**FIGURE 4:**
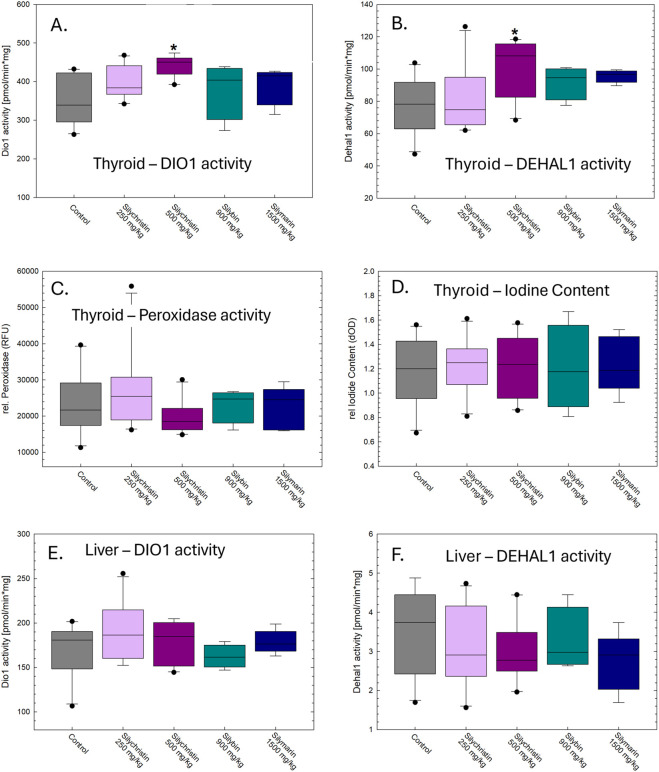
Mean (±SE) **(A)** deiodinase 1 (DIO1) and **(B)** dehalogenase activity in the thyroid gland **(A,B)** and liver **(C,D)** collected from animals 24-h after the last of nine daily doses of silychristin, silybin, and silymarin mixture. Significant increases in activity of DIO1 [F(4,34) = 4.45, p = 0.0054] and DEHAL1 [F(4,34) = 2.7, p = 0.05] were limited to the high-dose group. No changes in the activity of these enzymes were evident in the liver. Mean (±SE) relative peroxidase activity **(E)** and iodine content **(F)** were not altered in the thyroid gland (all p’s > 0.32] *p < 0.05 Dunnett’s t-test after significant main effect in ANOVA. n = 10/dose group for control and silychristin, n = 4 for silybin and five for silymarin.

In *ex vivo* examinations of metabolism enzymes from exposed animals, DIO1 and DEHAL1 activity were elevated in the thyroid glands extracted from animals treated with silychristin with significant increases over controls seen at the high-dose level for both enzymes (Figures 4A,B). This result was not attributable to general enzyme induction as, e.g., peroxidase activity was not changed (Figure 4C) and relative iodine content did not differ among any of the groups (Figure 4D), both being potential indicators of gland activation or changes in iodide transport or supply. Elevated activity of metabolizing enzymes was selective to the thyroid gland as no changes were seen in the liver in any treatment group (Figures 4E,F).

No change in the expression of transcripts encoding *Mct8* or related transmembrane transporters *Oatp1c1* or *Mct10* were detected in the choroid plexus of silychristin- or silybin-exposed rats. Nor were relative expression changes evident in deiodinase enzymes (*Dio1, Dio2,* and *Dio3*) or the distributor protein (*Ttr*). However, significant upregulation of both *Mct8* and *Oatp1c1* transcripts was observed in animals treated with silymarin (Table 2).

**TABLE 2:** Mean (±SE) fold-change for deiodinases, distributor, and transporter proteins in the choroid plexus of control vs. silychristin-, silybin-, and silymarin-exposed rats. A significant upregulation in thyroid hormone transmembrane transporters *Mct8* [F(4,33) = 3.33, p = 0.0212] and *Oatp1c1* [F(4,33) = 4.11, p = 0.0082] was observed and limited to the silymarin-treated animals. No significant changes in expression were seen for any other gene (all p’s > 0.24). *p < 0.05 Multiple comparison test using Dunnett’s t-test after significant main effect of ANOVA.

Treatment group	Dose	n	*Dio1*	*Dio2*	*Dio3*	*Ttr*	*Mct8*	*Oatp1c1*	*Mct10*
Control	0	10	1.06 ± 0.13	1.03 ± 0.10	1.19 ± 0.24	1.05 ± 0.12	1.03 ± 0.08	1.02 ± 0.06	1.03 ± 0.09
Silychristin	250	10	0.92 ± 0.12	0.86 ± 0.12	1.16 ± 0.16	1.11 ± 0.06	1.11 ± 0.08	1.30 ± 0.08	0.86 ± 0.10
Silychristin	500	10	0.99 ± 0.13	0.99 ± 0.12	0.99 ± 0.11	1.10 ± 0.05	1.03 ± 0.07	1.22 ± 0.08	0.84 ± 0.09
Silybin	900	4	0.88 ± 0.24	0.94 ± 0.32	1.49 ± 0.46	1.09 ± 0.05	1.23 ± 0.24	1.33 ± 0.15	1.09 ± 0.31
Silymarin	1,500	4	0.80 ± 0.13	0.79 ± 0.13	1.76 ± 0.13	1.21 ± 0.13	1.59 ± 0.13*	1.61 ± 0.13*	1.13 ± 0.13

## 4 Discussion

To bridge the gap between the reported *in vitro* activity in cell-based assays for TH transporter function (Johannes et al., 2016) and *in vivo* response, we conducted a 7-day dosing regimen in rats with the potent *in vitro* inhibitor of MCT8, silychristin, as a first attempt to explore potential effects from the pharmacological block of MCT8. Application of the corn-oil preparations of these compounds in the MCT8 *in vitro* assay showed the respective inhibitory potential of the preparations and confirmed the graded efficacy of silychristin, silybin, and silymarin to block MCT8-function (Johannes et al., 2016). Unlike lower serum T4 and elevated T3 of patients and mouse models with genetic MCT8 deficiencies (Di Cosmo et al., 2010; Schwartz and Stevenson, 2007; Friesema et al., 2010; Wirth et al., 2011), serum TH hormone profiles were unchanged in the rat following exposure to silychristin. However, increases in activity in DIO1 and DEHAL1 enzymes were observed in the thyroid gland and were limited to silychristin-exposed rats. No effects were seen in the liver, suggesting a specific vulnerability of the thyroid gland to MCT8 blockade. Our examination of transcripts encoding TH transmembrane transporters and metabolizing enzyme in the choroid plexus revealed no change from control following exposure to silychristin or silybin. However, surprisingly, significant increases in relative expression of *Mct8* and related transporter, *Oatcp1c1,* were evident in animals exposed to high doses of the flavonolignan extract, silymarin. Interpretation of this observation is challenging due to the limited information available on the constituents of the silymarin mixture. Our knowledge is restricted to the manufacturer-provided chemical analysis, which reported the share of silybin in this particular extract batch as 44.7%; the remaining content is largely unknown. As such, great uncertainty accompanies the possible genesis of the observed changes in transporter expression in the choroid plexus, yet various silymarin mixtures sold “over the counter” as detoxifiers and hepatoprotective agents are similarly devoid of chemical information on composition.

The largely negative effects observed *in vivo* highlight the complexities of translating *in vitro* action to responses *in vivo*. Certainly, pharmacokinetic and species-specific transporter profiles rank high among these factors, but elements of experimental study design also play a role—adult vs. developmental exposure scenarios, limited duration of dosing, bolus vs. more continuous exposure via food or water, and evaluations that were limited to a single point in time. The high cost to source and procure sufficient quantities of silychristin for *in vivo* dosing necessitated a short 7-day exposure regimen, limited achievable maximum dose levels, and unfortunately precluded an examination in a pregnancy model. Despite these limitations, this is the first study to address this important site of chemical interference of TH signalling *in vivo* and we did identify organ-specific increases in TH metabolizing enzyme activities in response to the inhibition of MCT8.

*Silychristin and thyroid gland:* Silychristin increased the activity of proteins and enzymes in the thyroid gland. MCT8 is located at the basolateral side of thyrocytes and is involved in TH efflux (Di Cosmo et al., 2010). In the murine *Mct8* knock out (KO) model, elevated levels of thyroidal TH content were detected, presumably as a consequence of limited release caused by the absence of this transporter. At 6–12 months of age, papillary hyperplasia emerged in the thyroid gland, paralleling changes detected in the thyroid morphology of AHDS-patients (Wirth et al., 2011). Prolonged interference with MCT8 function appears to be a requisite for gland dysmorphology. However, in the present study, a short-term reduction in TH release, caused by the silychristin-induced MCT8 inhibition, may have driven the observed elevations of metabolizing enzymes. Increases in DIO1 and DEHAL1 activity may be useful early local markers of altered transport function. These changes in the thyroid gland of silychristin-exposed rats are consistent with elevated activity of both DIO1 and DEHAL1 activity in hyperthyroid rats (Renko et al., 2022). Distinct from the hyperthyroid model, however, circulating TH levels were not yet changed in the present study, suggesting that the observed increases in gland metabolism are a response to glandular increases in TH levels, independent of systemic TH status. We postulate that a silychristin-dependent block of MCT8-mediated efflux from thyrocytes could result in an accumulation of TH within the gland, triggering an upregulation of both DIO1 and DEHAL1 activity.

In contrast to the gland, no changes in these metabolizing enzymes were observed in the liver of silychristin-exposed rats. Elevation of serum T3 in murine *Mct8* KO models was suggested to result in part from T3-mediated autoregulation of hepatic DIO1 metabolism (Liao et al., 2011), but targeted inactivation of hepatic DIO1 activity in *Mct8* KO mice did not change the serum TH profile (Wirth et al., 2015). Whether increased renal DIO1 activity or other alterations of TH economy are involved remains to be studied. The chemical interference of MCT8-mediated T3 transport in the present study was neither sufficiently strong or sustained enough to alter the activity of hepatic enzymes or raise serum T3. Recent *in vitro* studies have also revealed MCT8-mediated transport of iodotyrosines, TH biosynthesis byproducts that are substrates for DIO and DEHAL enzymes (Groeneweg et al., 2024). As such, augmented activities of both of these metabolizing enzymes that were limited to the thyroid gland may follow a rise in intracellular substrate levels from a reduction in MCT8-mediated TH efflux from thyrocytes (Gnidehou et al., 2004; Sun et al., 2017; Rokita et al., 2010). Further mechanistic studies are necessary to assess these possibilities.

*Chemistry and pharmacokinetics of isolated flavonoids:* Silymarin as a crude extract from milk thistle has a complex chemistry with many components that are challenging to purify, contributing to the low commercial availability and high cost of procurement of purified silychristin for *in vivo* studies. The composition of any given silymarin preparation is highly variable, dictated by factors including plant origin, cultivation, and processing (Kren and Valentova, 2022). Estimated levels of silychristin (10%–20%) and silybin (50%–60%) are present in the source material in addition to more than 10–12 major flavonolignan stereoisomers (Kren, 2021). Studies on pharmacokinetic properties of isolated constituents of flavonolignans are limited. In a review of flavonoids, Kren (2021), document pharmacokinetic differences across multiple species but conclude that rats are a useful and acceptable model for translation to humans. In one study in rats, purified silybin B, the most abundant constituent of silymarin, was reported to have a plasma half-life of 2.9 h and bioavailability of only 0.3% following a single oral gavage administration of 200 mg/kg (Marhol et al., 2015). Poor systemic oral bioavailability stemming from rapid conjugation and excretion by the liver (Marhol et al., 2015; Tvrdy et al., 2021) may underly the lack of effect of silychristin, silybin, and silymarin on serum TH in the present study.

*Species differences in kinetics and transporter expression:* In addition to pharmacokinetics, species differences in the spatio-temporal expression of TH transmembrane transporters and redundancy of transporter function in rodent models are well documented (Bernal et al., 2015; Groeneweg et al., 2020; Ueno et al., 2023). In *Mct8* KO mouse models, T3 uptake in the brain is severely blocked with only minor effects on T4 transport (Mayerl et al., 2014). Oatp1c1, a high-affinity transporter for T4 in rats and mice, is highly expressed on endothelial cells of the blood brain-barrier (Sugiyama et al., 2003; Mayerl et al., 2012). Oatp1c1 deficiencies in the mouse impair passage of T4 across the blood brain barrier and also induce a state of hypothyroidism in the brain (Mayerl et al., 2012). To emulate the neurological effects associated with MCT8 mutations in humans, however, genetic modification of *Mct8* transporter expression must be paired with silencing of genes encoding *Oatp1c1* transporter or *Dio2* enzyme (Morte et al., 2021). These studies indicate that, compared to humans, the redundancy in TH transmembrane transporters in rodents underlies their relative insensitivity to genetic interference with TH transport. This redundancy may also contribute to the lack of effect of flavonoids on serum TH and gene expression in the cerebral component of the present study.

*Genetic* vs*. chemical interference of transporter function:* Pharmacokinetic issues aside, short-term chemical interference with a potent MCT8 inhibitor is unlikely to lead to the level of sustained TH transport inhibition induced by gene-silencing approaches. While disruption of TH-mediated brain development is a concern for chemicals that interfere with MCT8-mediated transport, gestational and early postnatal deficiencies are required to induce this clinical profile in human patients and rodent models. Although the expression of MCT8 in many areas in the developing brain is high, it is transient, but expression is maintained in the choroid plexus of the adult primate and murine brain (Ueno et al., 2023; Wang et al., 2023). Therefore, in this preliminary short-term adult study, we reasoned that TH-sensitive transcripts of transmembrane transporter proteins and metabolizing enzymes in this component of cerebral tissue may be a promising readout to assess the presence of local changes in transporter-mediated concentrations of TH. We did not detect changes in the relative expression of TH-dependent genes in this brain tissue following exposure to silychristin or silybin. However, we did see a significant upregulation of both *Mct8* and *Oatpc1c1* transcripts in silymarin-exposed rats. Despite the higher dose of silymarin relative to the other flavonolignans (1,500 vs. 500 and 900 mg/kg, respectively for silymarin, silychristin and silybin), this effect is not readily attributed to the silychristin or silybin components of silymarin. Comparison of *in vitro* potency of the dosing solutions in an *in vitro* MCT8 inhibition assay IC50 estimates are ∼0.1 µM for silychristin and 5 µM for silymarin but approach 100 µM for silybin. Even if concentrations of silychristin present in the silymarin dosing solution were as high as 20% (EMA/HMPC, 2018) (as estimated by the European Medicines Agency, 2018), a 3-fold higher administered dose would still fall short of the silychristin concentration for which no change in expression of these genes was observed. It is possible that interactions of flavonolignans in the silymarin mixture may have a differential effect on pharmacokinetics, or actions of other components of the mixture unrelated to TH transporter function may underlie these observations. Based only on a sample size of four silymarin-exposed rats, replication of these findings is also required.

In summary, identification of chemicals with biological activity using high-throughput *in vitro* screening assays is an important tool in the arsenal to detect and prioritize testing of environmental chemicals for potential health hazards. Validation and contextualization of positive *in vitro* findings require assessment in more complex, integrative test systems. To our knowledge, this report represents the first *in vivo* study examining the potential impact of a potent and selective *in vitro* MCT8 inhibitor on the TH system. Increases in DIO1 and DEHAL activity were identified in the thyroid glands of silychristin-exposed animals and may reflect a compensatory upregulation in response to an accumulation of thyroid TH due to the block of MCT8-mediated efflux from thyrocytes. These findings also suggest that local enzyme activities have the potential to serve as indicators of organ-specific effects of some classes of thyroid hormone system-disrupting chemicals. Although pharmacokinetic properties of silychristin and redundancy in transporter expression may attenuate consequences of MCT8 blockage in rodents, it is conceivable that these chemicals may demonstrate a considerably more pronounced effect in humans.

## Data Availability

The raw data supporting the conclusions of this article will be made available by the authors, without undue reservation.
